# Noninvasive imaging of rat-derived microglia and its reactivity to inflammatory molecules via acoustic impedance microscopy

**DOI:** 10.1007/s10396-023-01379-8

**Published:** 2023-11-16

**Authors:** Christine Li Mei Lee, Pey Shin Yap, Kiyoshi Umemura, Taichi Shintani, Kazuto Kobayashi, Naohiro Hozumi, Sachiko Yoshida

**Affiliations:** 1 Department of Applied Chemistry and Life Science, Graduate School of Engineering, Toyohashi University of Technology, Toyohashi, Aichi 441-8580 Japan; 2 Department of Electrical and Electronic Information Engineering, Graduate School of Engineering, Toyohashi University of Technology, Toyohashi, Aichi 441-8580 Japan; 3 Honda Electronics Co., Ltd., Toyohashi, Aichi 441-3147 Japan

**Keywords:** Scanning acoustic microscope, Acoustic impedance, B-mode, C-mode, Cell observation

## Abstract

**Purpose:**

Microglia, the brain's immune cells, play important roles in neuronal differentiation, survival, and death. The function of microglia is deeply related to the morphologies; however, it is too complex to observe conventionally and identify the condition of living microglia using optical microscopes. Herein, we proposed a new method to observe living cultured microglia and their reactivity to inflammation via the acoustic impedance mode of a scanning acoustic microscope.

**Methods:**

Primary cultured microglia collected from rat pups exposed to acetamiprid, an insecticide, in utero were observed with both acoustic interface impedance mode (C-mode) and transparent three-dimensional impedance mode (B-mode).

**Results:**

We characterized microglia into four types based on the results obtained from acoustic impedance, cytoskeletal information, and laser confocal imaging. Biphasic acoustic observation using B-mode and C-mode gave us information regarding the dynamic morphologies of living microglia treated with adenosine triphosphate (ATP) (600 μmol/L), which reflects distress signals from inflamed neurons. Acetamiprid exposure induced microglia response even in the neonatal period. ATP stimulus altered the shape and thickness of microglia with a change in the bulk modulus of the cell. Three-dimensional alteration with ATP stimulus could be observed only after biphasic acoustic observation using B-mode and C-mode. This acoustic observation was consistent with confocal observation using anti-Iba-1 and P2Y12 immunocytochemistry.

**Conclusion:**

This study demonstrated the adequacy of using a scanning acoustic microscope in analyzing microglia's shape, motility, and response to inflammation.

**Supplementary Information:**

The online version contains supplementary material available at 10.1007/s10396-023-01379-8.

## Introduction

Two-dimensional (2D) scanning acoustic microscopy (SAM) is a useful tool for the direct observation of biological tissue using high-frequency ultrasonic waves [1–8]. One of the advantages of this microscopy method is observing the elastic properties of living cells without undergoing the histochemical staining process [6]. In other words, the ability to observe living cells without fixation or antibodies. This is important as fixation causes the cells to die, hence disabling the observation of dynamic biological processes. Acoustic observation has demonstrated that the application of drugs, such as anticancer drugs, to cells can also reduce and change the trends of acoustic impedance [3]. SAM allows intracellular observation along the depth direction (B-mode). Hozumi et al. [1] reported having converted the reflection intensity from the internal region of glial cells into the distribution of acoustic impedance via deconvolution in the frequency domain, thus obtaining a three-dimensional (3D) cross-sectional view of the cell.

The present study investigated 3D observation and estimation of aggression of living microglia (MG) treated with an insecticide prenatally. MG, the target cell of this research, are a type of neuroglia, others being astrocytes and oligodendrocytes. MG are the primary immune cell in our central nervous system (CNS), accounting for 10–15% of cells in the brain [9]. Their primary function is to maintain the delicate homeostasis of the brain. As the brain's primary immune cells, they are actively involved when neuroinflammation occurs. MG express a variety of motilities to carry out their function under physiological and pathological environments. In the physiological environment, they are actively scavenging and surveying the brain for abnormalities. MG are conventionally assigned as M0, M1, or M2 MG [10–16]. The M0 phenotype or “resting state” is actively scavenging and surveying the brain for abnormalities. Alternatively, in a pathological environment, it is activated and migrates toward the trauma site, turning into phagocytotic cells to engulf damaged cells. The M1 phenotype is a common way to characterize this proinflammatory function of MG. Another morphology of MG in between these two environments is the M2 phenotype, in which MG are anti-inflammatory. They protect surrounding cells from inflammation. However, the M0, M1, and M2 phenotypes and the term “activated” MG are oversimplified and may no longer be viable ways to classify these complicated and varied MG morphologies [17]. Therefore, using SAM with C-mode and B-mode acoustic imaging paired with confocal microscopy may elucidate how to study and classify various living MG motilities.

For MG to carry out their function, they express a lot of protein on their surface, acting as receptors to detect even small changes in the brain condition. Receptors that detect purines, such as adenosine triphosphate (ATP), are the focus of this research. ATP is released into the extracellular environment by inflamed cells [18, 19]. When MG detect this distressed cell signal in ATP, they respond to it to perform their function [9].

Aside from ATP as stimulation for inflammation, acetamiprid (ACE) was also used. ACE, a type of insecticide from the group neonicotinoid, mimics the nicotine chemical structure to bind to the nicotinic acetylcholine receptors. Neonicotinoids have been linked to the colony collapse disorder of bees [20, 21], while the effects of neonicotinoids on humans are considered safe as they are thought to be highly specific to targeted pests; however, some reports suggest otherwise [22–29]. ACE may have the potential to cause damage to humans, especially to the developing brain. Therefore, further research on the usage of ACE is vital.

Although extensive research is performed on the function of MG, studies on its morphology are still lacking and difficult to achieve with the current observation method. The conventional methods of observing MG are via optical and confocal microscopes. Although these methods are widely used, they are insufficient to study the dynamicity of MG. An optical microscope using phase-contrast imaging can only provide information on the shape of the MG without further internal cytoskeleton distribution details. A confocal microscope requires a fixing and staining process, causing cells to no longer be viable. Observing chemotaxis in MG as immune cells in the brain is crucial for a better understanding of neurological diseases. This paper proposed observing living MG and their morphological changes and dynamicity in response to inflammation via the scanning acoustic microscope. We further confirmed MG observation and dispersion of MG-specific markers Iba-1 and P2Y12 via a confocal microscope.

## Materials and methods

### MG cell preparation

**Fig. 1:**
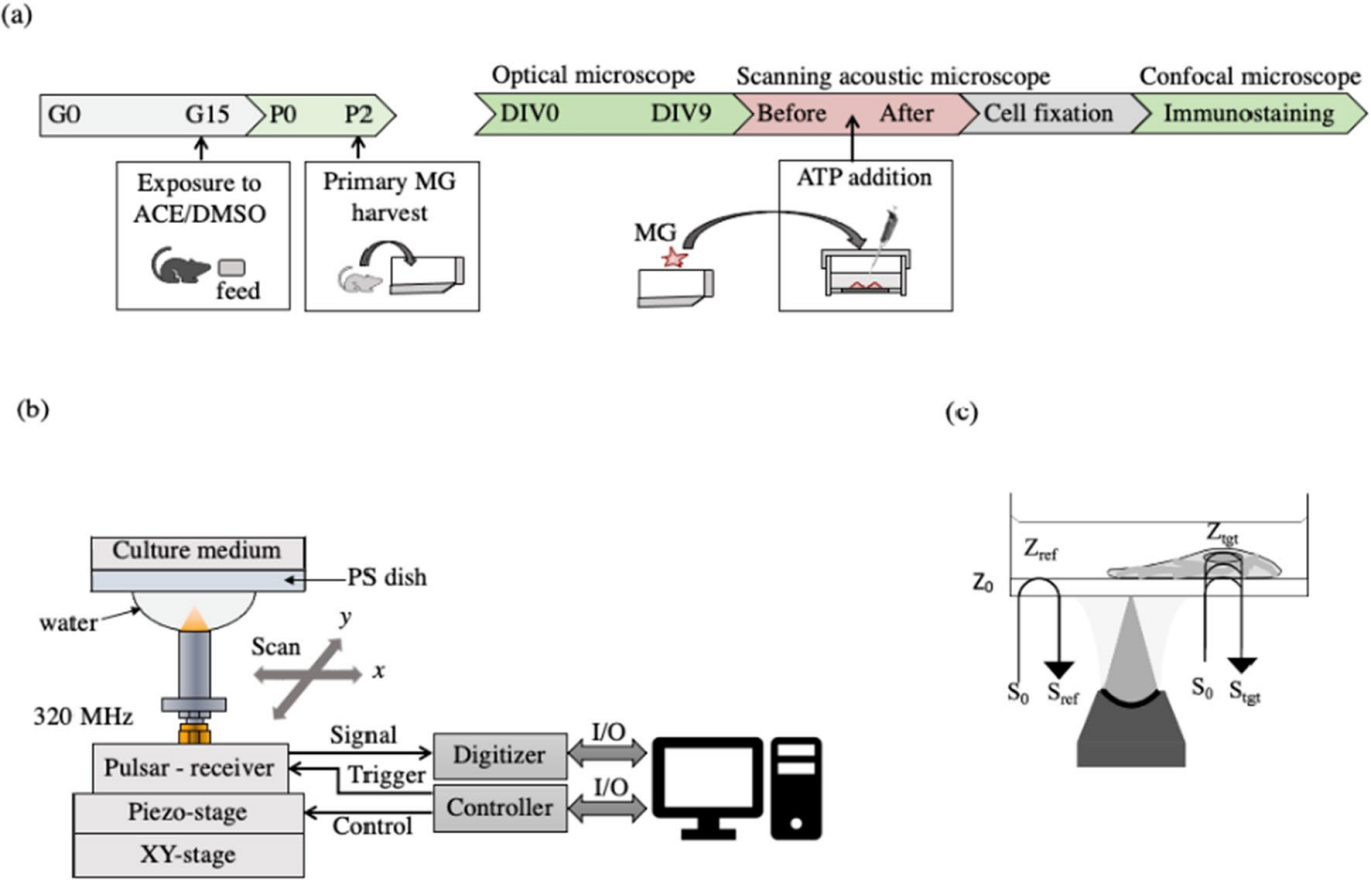
Schematic diagram of the experiment setup. **a** Flow chart of methodology. *G* Gestation, *P* postnatal, *DIV* days in vitro, *ACE* acetamiprid, *MG* microglia. **b** Measurement system of scanning acoustic microscope. PS dish: Polystyrene dish. **c** Illustration of acoustic impedance

A flow chart of the methodology is shown in Fig. 1a. Wistar rats (Japan SLC, Inc., Japan) were used as experimental animals in this study. ACE (Fujifilm Wako Pure Chemical, Japan) 40 mg/kg body weight [30] was administered to pregnant Wistar rats on gestation day 15 (G15) through the feed. Another set of control rats were given dimethyl sulfoxide (DMSO), used as a solvent for ACE, and prepared using the same preparation method as mentioned above. Glial cells from the cerebellum of female pups born on postnatal day 2 (P2) were cultivated in an angled neck culture flask using glia-selective culture medium composed of Dulbecco’s modified eagle medium (DMEM) supplemented with 0.4% d-glucose, 10% newborn calf serum (NBCS), 0.02% kanamycin, and primary culture medium, changed once every 2 days or when needed. All cells were cultured in a 5% CO_2_ incubator at 37 °C. MG were seeded on a poly-l-ornithine-coated polystyrene film dish (Honda Electronics Co., Ltd., Japan) with 50-μm thickness for acoustic and optical observation. Cultured MG were stimulated with 600 μmol/L adenosine triphosphate (ATP), which reflects neuronal injury signals [31]. ATP-stimulated MG were observed consecutively for 4 h using SAM.

## Acoustic cell observation and calculation

The setup for the acoustic measurement system is shown in Fig. 1b. A focused pulsed ultrasonic wave with a center frequency of about 320 MHz (range 200–400 MHz) was transmitted from the ultrasonic transducer with an aperture diameter of 1.04 mm and a focal length of 0.69 mm. The transducer was controlled by a piezo stage with a resolution of up to 4 µm with a scanning area of 180 × 180 μm and 200 × 200 points (*x* and *y* resolutions). Sampling intervals were taken at 200 ps with an average of 8.

Calculation of the acoustic impedance, of which details have been described in previous papers [1, 2], is illustrated in Fig. 1c. For C-mode, the acoustic impedance is obtained by comparing reflection intensity directly with a reference signal. Briefly, ${S}_{tgt}(t)$ is the signal reflected from the target when ${S}_{0}(t)$ is transmitted, where $t$ is time. The impulse response from the sample is:

1 $$\Gamma \left(\omega \right)=\frac{{S}_{tgt}(\omega )}{{S}_{0}(\omega )},$$

where ${S}_{0}(\omega )$ and ${S}_{tgt}(\omega )$ are the Fourier transforms of ${S}_{0}(t)$ and ${S}_{tgt}(t)$, respectively. ${S}_{0}(\omega )$ in Eq. (1) can be calculated from:

2 $${S}_{\mathrm{ref}}=\frac{{Z}_{\mathrm{ref}}-{Z}_{0}}{{Z}_{\mathrm{ref}}+{Z}_{0}}{S}_{0},$$

where ${S}_{\mathrm{ref}}$ is the response from the reference material, of which acoustic impedance is known. For B-mode, neglecting the attenuation through each layer, the signal can be successively converted to acoustic impedance ${Z}_{i}$ in the depth direction by referring to ${Z}_{0}$, which is the acoustic impedance of the substrate.

3 $${\Gamma }_{i}=\frac{q({t}_{i})}{q({t}_{0}){\prod }_{0}^{i-1}{T}_{j}},$$

4 $${Z}_{i+1}=\frac{1+{\Gamma }_{i}}{1-{\Gamma }_{i}}{Z}_{i},$$

5 $${T}_{i}=\frac{4{{Z}_{i}Z}_{i+1}}{{{(Z}_{i+1}+{Z}_{i})}^{2}},$$

where ${\Gamma }_{i}$ is the reflection coefficient at each interface, and $q(t)$ is the inverse Fourier transform of $\Gamma \left(\omega \right)$ that can be perceived as the apparent reflectance as seen from the substrate, including all the effects of reflections from the interface and target. ${t}_{i}$ corresponds to each sampling time, and the product of the sampling interval (${t}_{i+1}-{t}_{i}$) and sound speed corresponds to the thickness of each fragmental layer. A 3D acoustic impedance image can be created by scanning the transducer along *x*–*y* directions. Acoustic impedance is calculated based on the average taken from three points across the cell.

### Fluorescent immunostaining

To understand why acoustic impedance increased or decreased in the filopodia of MG, we stained targeted MG with MG-specific Iba-1 and P2Y12 antibodies and observed them under confocal microscopy. Iba-1, short for ionized calcium-binding adapter molecule, is a protein involved in actin bundling to provide structure and shape to filopodia and lamellipodia of MG. P2Y12 is an ADP/ATP receptor and is specifically expressed in MG in the CNS.

After being seeded on a poly-l-ornithine-coated polystyrene film dish, samples were fixed with 4% paraformaldehyde (PFA) solution in PBS for 15 min and washed thrice with 1XPBS. Samples blocked with 0.1% Triton X-100 and 5% goat serum were stained with primary antibodies for anti-Iba-1 (mouse, Fujifilm Wako Pure Chemical) and anti-P2Y12 (rabbit, Thermo Fisher) and with secondary antibodies for anti-mouse IgG labeling with Alexa488 and anti-rabbit IgG labeling with Texas Red (Thermo Fisher).

### Statistics

All value ranges are given as mean ± standard error of the mean (SEM) and calculated from three independent samples. Statistical significance was estimated by paired *t* *test*. P values less than 0.05, 0.01, and 0.001 were considered significant, highly significant, and very highly significant, respectively, for all the tests.

## Results

### ACE-exposed MG showed secondary immune response, while DMSO-exposed MG showed primary immune response with ATP stimulation

**Fig. 2:**
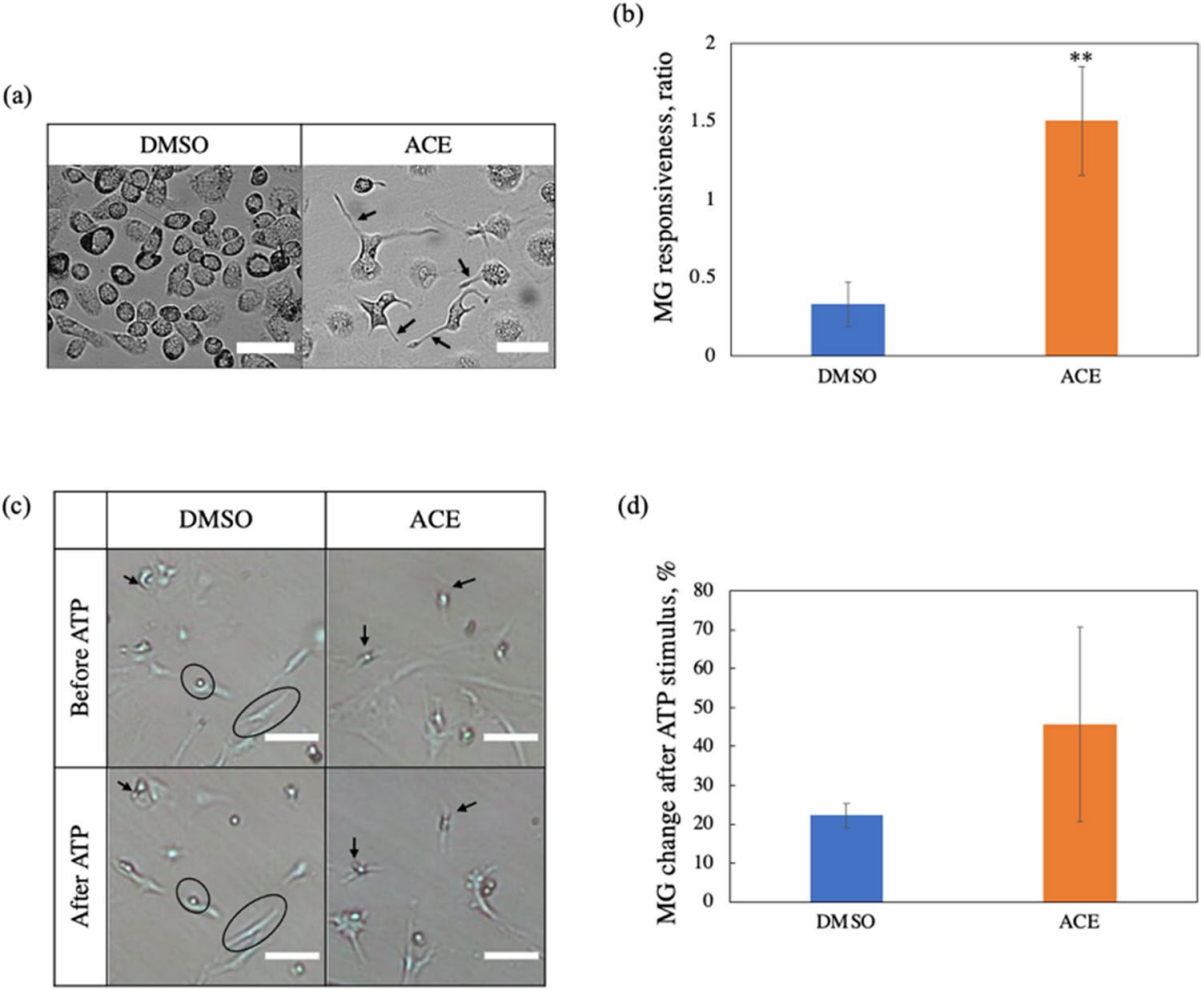
**a**, **b** MG states during cell culture. **a** Observation via optical microscope. **b** Responsiveness of MG to treatment (*p* = 0.0031). **c**, **d** MG states before and after ATP stimulus. **c.** Observation via optical microscope. **d** Responsiveness of MG to ATP stimulus. Scale bar 50 μm

Primary MG were cultured from ACE-exposed pups, while DMSO-exposed MG were cultured as a control group. DMSO is used as a solvent for ACE. During cell culture, in DMSO-exposed MG, MG were not as immune responsive (Fig. 2a left and b); while in ACE-exposed MG, MG were highly significantly immune responsive with the formation of filopodia (arrows, Fig. 2a right and b). MG immune responsiveness ratios were calculated as the ratio of filopodia-forming cells to rounded cells.

To induce a secondary immune response, both MG cell cultures were stimulated with ATP to mimic distress signals from inflamed neurons. After ATP stimulus, ACE-exposed MG have a higher tendency of an immune response compared to DMSO-exposed MG (arrows indicate changes, circles indicate no changes, Fig. 2c and d). MG changes after ATP stimulus were calculated as shape change over total calculated cells in percentage.

### C-mode acoustic imaging showed MG motility and acoustic impedance

**Fig. 3:**
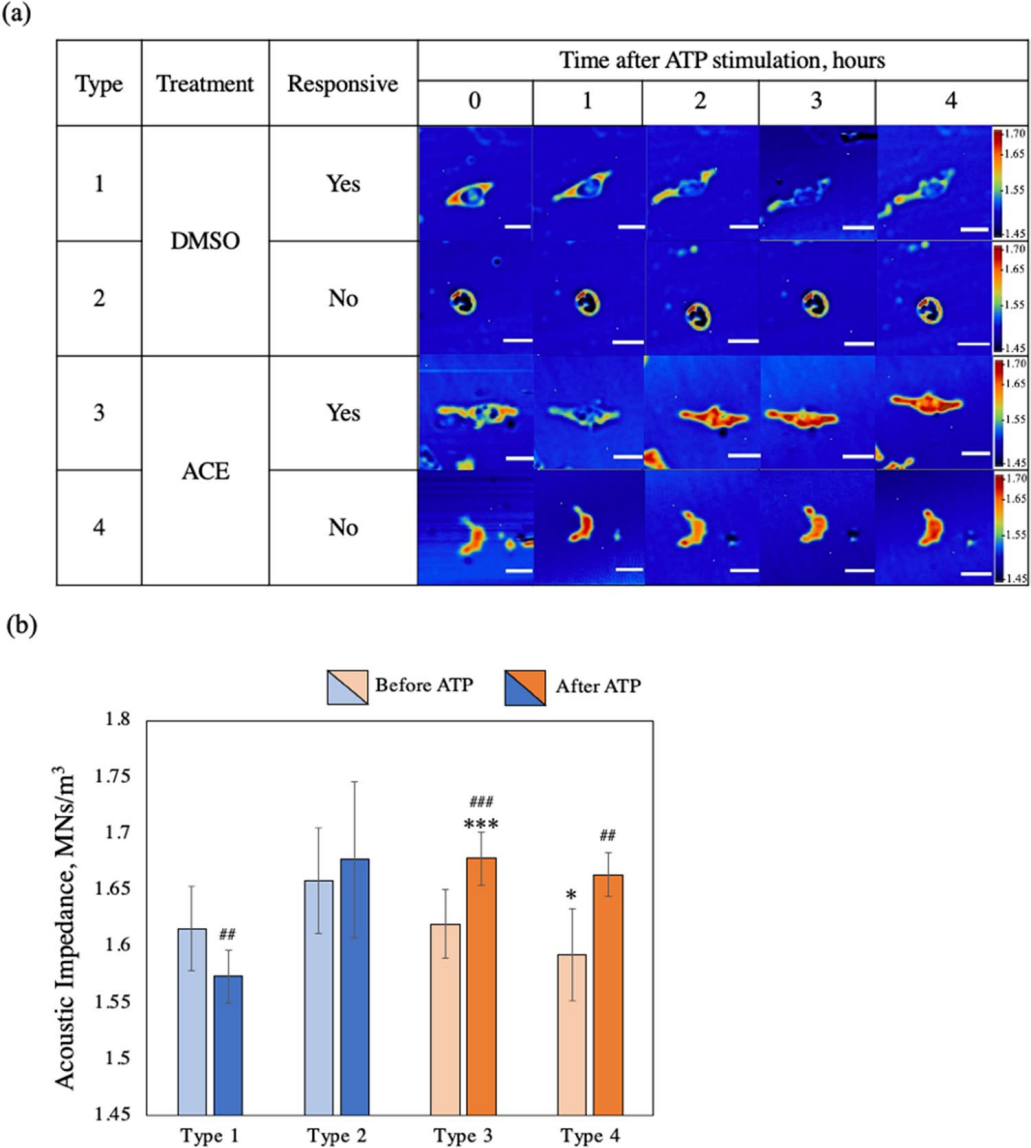
**a** Observation of MG response to ATP stimulus via C-mode acoustic impedance imaging over a 4-h period. Scale bar 50 μm. **b** Acoustic impedance value of MG before and after 4-h ATP stimulus. Before and after ATP stimulus; highly significantly decreased in type 1 (^##^*p* = 0.0058), very highly significantly increased in type 3 (^###^*p* = 0.0007), and highly significantly increased in type 4 (^##^*p* = 0.0051). *p < 0.05 vs DMSO, ***p < 0.001vs DMSO, ^##^*p* < 0.01 vs before ATP, ^###^*p* < 0.001 vs before ATP. Calculated from three different samples

MG were observed every hour for 4 h after ATP stimulus using C-mode acoustic impedance with a scanning area of 180 × 180 μm, scanned with 200 × 200 scanning points (Fig. 3). The round dots surrounding MG were artifacts and were ignored in this study. The shapes of MG were observed clearly on C-mode acoustic imaging.

We observed four types of MG: two each from DMSO and ACE-exposed MG. DMSO-exposed MG, when immune responsive and not immune responsive with ATP, were each characterized as types 1 and 2 MG, respectively. On the other hand, when immune responsive and not immune responsive to ATP, ACE-exposed MG were characterized as types 3 and 4, respectively. Responsiveness of MG was noted as changes in terms of shape or formation of filopodia based on macroscopic observation on C-mode imaging.

In type 1, acoustic impedance decreased significantly after ATP stimulus (from 1.616 to 1.573 MNs/m^3^). The filopodia in these MG were also extended. In type 2, acoustic impedance and morphology remained unchanged (from 1.658 to 1.677 MNs/m^3^), with no filopodia formation.

Types 3 and 4 MG were already immune responsive during ACE exposure. Type 3 showed a secondary immune response whereby the MG motility were observed after ATP stimulus. Type 4 MG remained in primary immune response and did not change morphology with ATP stimulus. After ATP stimulus, the acoustic impedance of MG were very highly significantly increased for type 3 (from 1.620 to 1.678 MNs/m^3^) and highly significantly increased for type 4 (from 1.593 to 1.664 MNs/m^3^).

### B-mode acoustic imaging of MG showed the thickness of MG before and after ATP stimulus

**Fig. 4:**
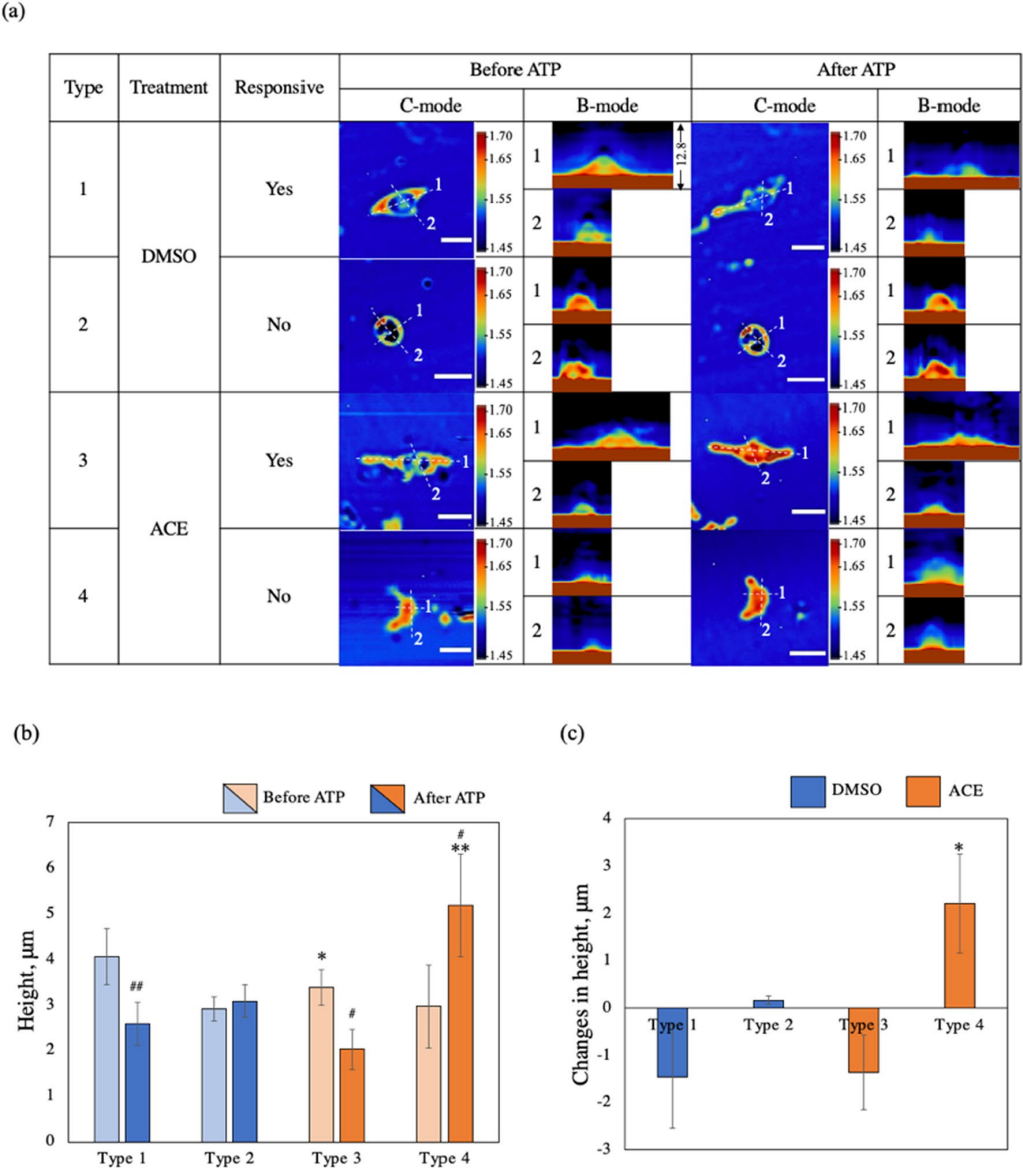
**a** Thickness of MG along cross-sections 1 and 2 via C- and B-mode acoustic impedance imaging before and after 4-h ATP stimulus. Scale bar 50 μm. **b** Height of MG before and after ATP. Before and after ATP stimulus; highly significantly thinner in type 1 (^##^*p* = 0.0056), significantly thinner in type 3 (^#^*p* = 0.0110), and significantly thicker in type 4 (^#^*p* = 0.0282). **c** Changes in height of MG. *p < 0.05 vs DMSO, **p < 0.01vs DMSO, ^#^*p* < 0.05 vs before ATP, ^##^*p* < 0.01 vs before ATP. Calculated from three different samples

We calculated B-mode images based on a previously described method [2] from the C-mode acoustic impedance of the MG cell along cross-section lines 1 and 2 (Fig. 4a**)** and summarized the height and changes in height of MG before and after ATP stimulus in Fig. 4b and c**,** respectively. The height of MG was measured from the acoustic impedance image and compared with deconvolution and grayscale (analyzed with Image J) images (Supplementary file), which have a clearer boundary of the cell and cultured medium.

Type 1 MG became highly significantly thinner after ATP stimulus (decreased on average 1.465 μm). Type 2 MG were not immune responsive, and the thickness of MG roughly did not change (increased on average 0.164 μm).

Type 3 MG showed a secondary immune response and the MG became significantly thinner after ATP stimulus (decreased on average 1.357 μm). Type 4 MG did not respond to ATP stimulus but were already in a primary immune response. Interestingly, these MG became significantly thicker (increased significantly on average 2.215 μm).

### Confocal imaging of MG showed Iba-1 and P2Y12 dispersion across MG

**Fig. 5:**
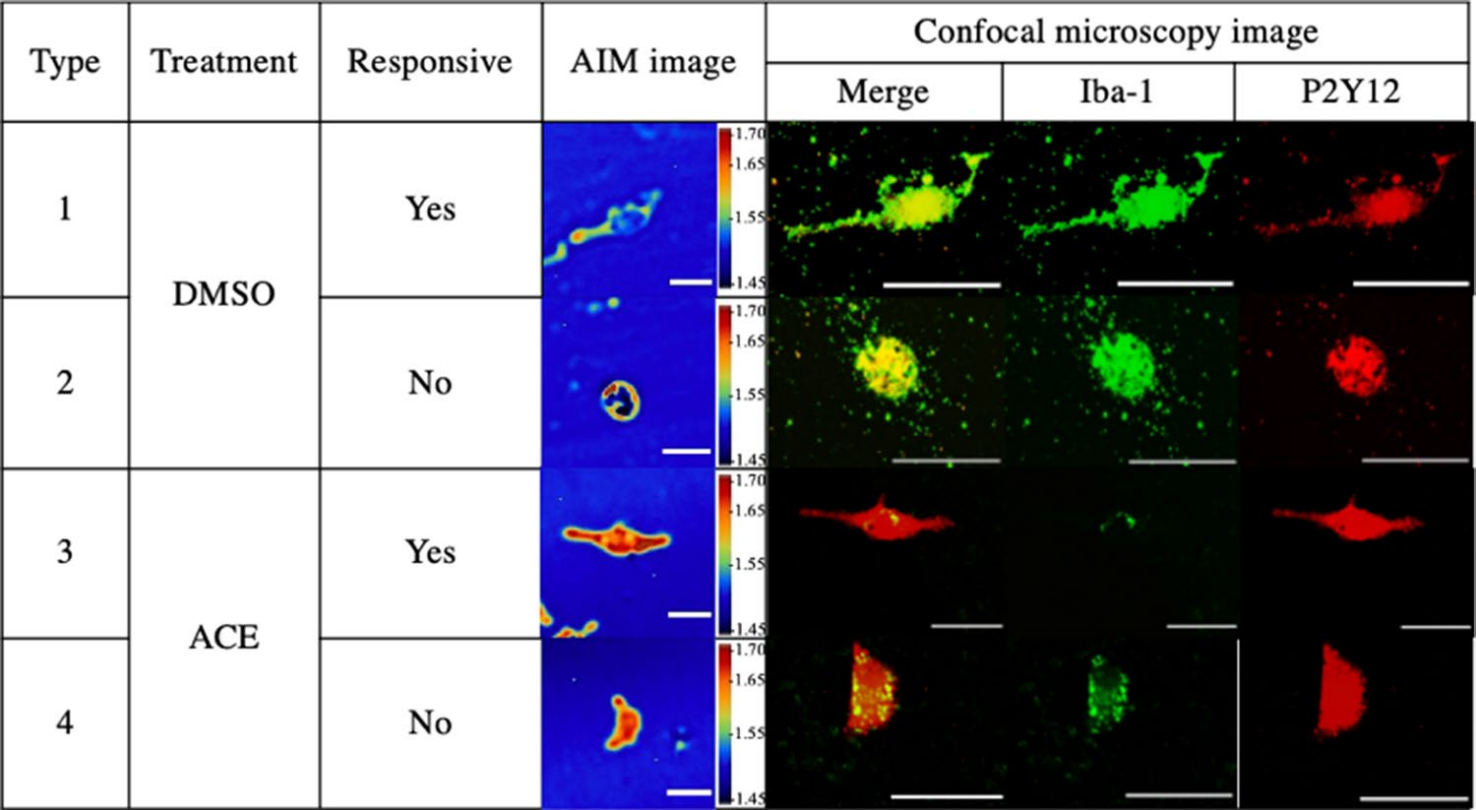
Observation of MG receptors Iba-1 and P2Y12 via confocal microscopy after 4-h ATP stimulus. Scale bar 50 μm

The confocal microscopy results showed that both Iba-1 and P2Y12 were expressed in all MG (Fig. 5). Interestingly, Iba-1 expressions in ACE-exposed types 3 and 4 MG were lesser than in DMSO-exposed types 1 and 2 MG**.** However, in ACE-exposed MG, type 4 MG showed higher expression of Iba-1 compared to type 3 MG.

On the other hand, P2Y12 was more highly expressed in ACE-exposed types 3 and 4 MG compared to DMSO-exposed types 1 and 2 MG.

## Discussion

Through C-mode acoustic imaging, we were able to observe consecutively for 4 h the changes in acoustic impedance and the morphology of MG without utilizing any cell fixation or antibodies. In addition, we observed the thickness of MG during motility with B-mode acoustic imaging.

A softer cytoskeleton shows faster actin turnover [32]. In type 1 MG, acoustic impedance decreased after ATP stimulation, which indicated the cytoplasm of MG becoming softer, leading to higher actin turnover. This led to filopodia and lamellipodia formation, which indicated phagocytosis as the MG reaction after ATP stimulus that we observed. These MG were dynamic to carry out a phagocytic function, spreading their filopodia in response to ATP stimulus, causing the height of MG to decrease. In type 2, the MG were not reactive to ATP stimulus. We speculated that these unreactive MG were very rigid and not dynamic; there were no differences in the height and acoustic impedance of MG.

Interestingly, Iba-1 was not highly expressed in ACE-exposed MG. Agarwal et al. [33] reported that primary phagocytosis resulted in increased reactive oxygen species (ROS) and decreased actin turnover, which mediated cytoplasmic stiffening in macrophages, resulting in impaired secondary phagocytic function. Many reports have shown that ACE causes an increase in ROS in cell culture [34, 35]. Similarly, in types 3 and 4 MG, we hypothesize that during ACE exposure, increased ROS resulted in MG stiffening, as observed by the increased acoustic impedance after ATP stimulus. In type 3 MG, morphology-wise, we observed MG widen around the cell body instead of extending their already-formed filopodia, which might have led to an impaired secondary phagocytotic behavior, as proposed by Agarwal et al. Additionally, Iba-1 is involved in membrane ruffling and phagocytosis in MG [36]. We suggest that ACE-exposed MG having an impaired secondary phagocytotic function caused low expression of Iba-1. However, in type 4 MG, instead of forming filopodia, these MG became thicker in height, as observed on B-mode acoustic imaging. It is unclear why the response to inflammation differs from that of type 3. The addition of ATP might influence the pH level of cells. MG nearer to ATP addition may respond differently to MG further from ATP addition. In the future, we can mark the ATP addition point to elucidate this hypothesis.

Immune cell aggressiveness is a black box for host bodies. Aggressive MG induce heavy vasculitis with cytokine storm and lead to brain inflammation. Our study showed that B-mode observation of living immune cells could identify immune cell aggressiveness.

## Conclusion

Understanding how living MG are responsive to a certain inflammation may pave the way to discovering future drugs to counteract the inflammation. This study demonstrated the adequacy of using a scanning acoustic microscope in analyzing MG shape, motility, and response to inflammation. The B-mode cross-sectional acoustic impedance image analysis with deconvolution in time and frequency domains proposed in the previous study allowed observation of the thickness of MG when responding to ATP stimulus. In the future, stacking the B-mode images into a 3D view will provide a better insight into the internal structure of MG.

### Supplementary Information

Below is the link to the electronic supplementary material.

Supplementary file1 (DOCX 406 KB)
