# Supplementary material for: Noninvasive imaging of rat-derived microglia and its reactivity to inflammatory molecules via acoustic impedance microscopy
Source: J Med Ultrason (2001). 2023 Nov 16;51(1):29–37. doi: 10.1007/s10396-023-01379-8 (PMC10803564; doi:10.1007/s10396-023-01379-8)
Supplement: Supplementary file 1 — Supplementary file1 (DOCX 406 KB) [file 10396_2023_1379_MOESM1_ESM.docx]

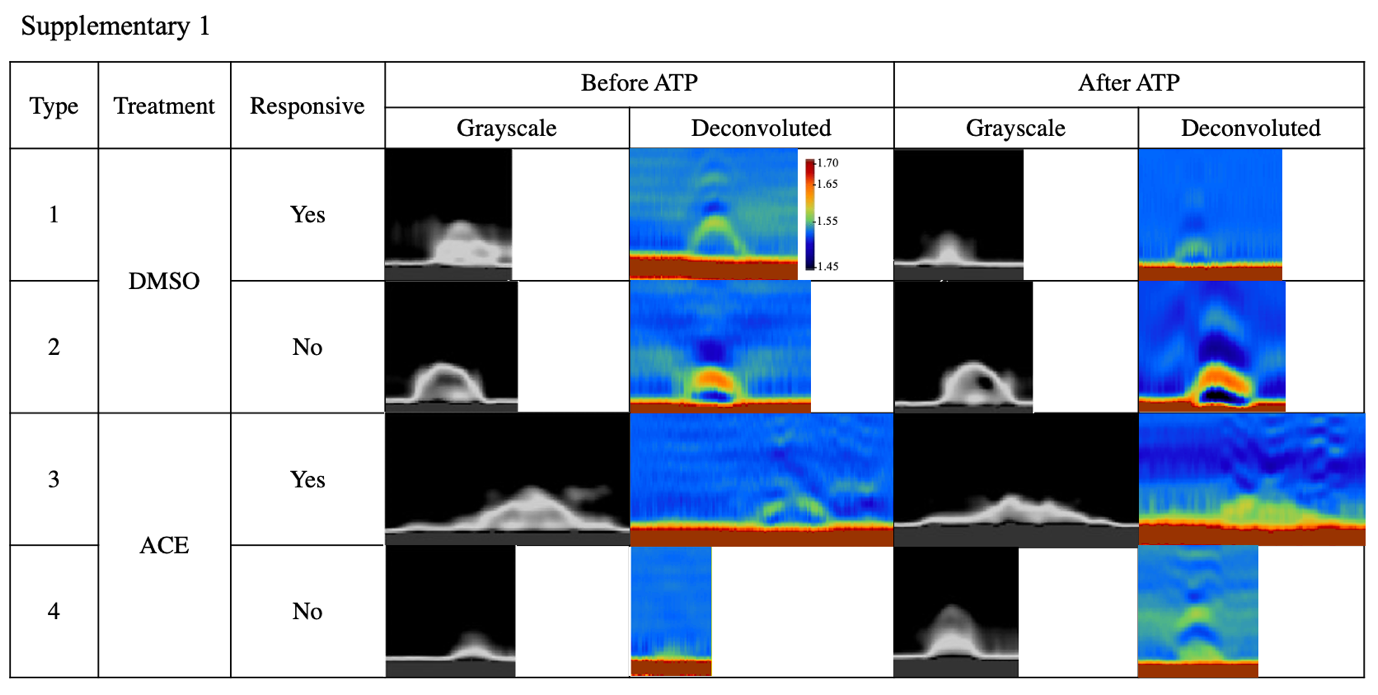


**Supplementary 1.** Grayscale (from acoustic impedance images) and deconvolution images of B-mode imaging before and after 4-hour ATP stimulus.
